# *De novo* and comparative transcriptomic analysis explain morphological differences in *Panax notoginseng* taproots

**DOI:** 10.1186/s12864-021-08283-w

**Published:** 2022-01-31

**Authors:** Lifang Yang, Hanye Wang, Panpan Wang, Mingju Gao, Luqi Huang, Xiuming Cui, Yuan Liu

**Affiliations:** 1 Faculty of Life Science and Technology, Kunming University of Science and Technology, Kunming, 650000 China; 2 Wenshan University, Wenshan, 663000 China; 3 National Resource Center for Chinese Materia Medica, Chinese Academy of Chinese Medical Sciences, Beijing, 100700 China; 4 Key Laboratory of Panax notoginseng Resources Sustainable Development and Utilization of State Administration of Traditional Chinese Medicine, Kunming, 650000 China; 5 Yunnan Provincial Key Laboratory of Panax notoginseng, Kunming, 650000 China; 6 Kunming Key Laboratory of Sustainable Development and Utilization of Famous-Region Drug, Kunming, 650000 China; 7 Sanqi Research Institute of Yunnan Province, Kunming, 650000 China

**Keywords:** *Panax notoginseng*, Taproot, Phenotypic differences, *De novo* assembly transcriptome, Differentially expressed genes

## Abstract

**Background:**

*Panax notoginseng* (Burk.) F. H. Chen (PN) belonging to the genus *Panax* of family Araliaceae is widely used in traditional Chinese medicine to treat various diseases. PN taproot, as the most vital organ for the accumulation of bioactive components, presents a variable morphology (oval or long), even within the same environment. However, no related studies have yet explained the molecular mechanism of phenotypic differences. To investigate the cause of differences in the taproot phenotype, *de novo* and comparative transcriptomic analysis on PN taproot was performed.

**Results:**

A total of 133,730,886 and 114,761,595 paired-end clean reads were obtained based on high-throughput sequencing from oval and long taproot samples, respectively. 121,955 unigenes with contig N50 = 1,774 bp were generated by using the *de novo* assembly transcriptome, 63,133 annotations were obtained with the BLAST. And then, 42 genes belong to class III peroxidase (PRX) gene family, 8 genes belong to L-Ascorbate peroxidase (APX) gene family, and 55 genes belong to a series of mitogen-activated protein kinase (MAPK) gene family were identified based on integrated annotation results. Differentially expressed genes analysis indicated substantial up-regulation of *PnAPX3* and *PnPRX45*, which are related to reactive oxygen species metabolism, and the *PnMPK3* gene, which is related to cell proliferation and plant root development, in long taproots compared with that in oval taproots. Furthermore, the determination results of real-time quantitative PCR, enzyme activity, and H_2_O_2_ content verified transcriptomic analysis results.

**Conclusion:**

These results collectively demonstrate that reactive oxygen species (ROS) metabolism and the *PnMPK3* gene may play vital roles in regulating the taproot phenotype of PN. This study provides further insights into the genetic mechanisms of phenotypic differences in other species of the genus *Panax*.

**Supplementary Information:**

The online version contains supplementary material available at 10.1186/s12864-021-08283-w.

## Introduction

*Panax notoginseng* (Burk.) F. H. Chen (PN) is a perennial herb belonging to the Araliaceae family. PN taproot, as the most vital organ for the accumulation of bioactive components, is a raw material of many famous patented Chinese medicines, including Yunnan Baiyao, Xuesaitong, and Compound Danshen Dripping Pills [1, 2]. The main bioactive components isolated from PN taproots include ginseng saponins, notoginsenoside, and dencichine [2, 3]. Extensive pharmacology studies have shown that PN prevents cardio- and cerebrovascular disease, has anti-inflammatory effects, and aids immune regulation [4, 5]. Xuesaitong, produced from the total saponins extracted from PN taproots, is in broad clinical use for the prevention and treatment of hyperlipidemia, coronary heart disease, stroke sequelae, and other chronic diseases, and is particularly favored by patients. In general, the growth rate of PN is relatively slow in the wild, and even with good field management it takes at least three years to grow and accumulate sufficient bioactive components in its taproots before they can be harvested and used as a medicinal material. However, in the process of PN cultivation, there has been a perplexing phenomenon wherein there are obvious differences in the root phenotype during growth, even when many PN plants are grown in the same environment. Specifically, some of them present long strip-like ginseng (long taproot of PN: LPN), whereas others are oval-like (oval taproot of PN: OPN); they are called “Luobo qi” or “Chang qi” and “Geda qi” or “Tuan qi,” respectively.

To date, many studies have focused on the biosynthetic pathways, whole-genome expression profile, and pharmacological effects of the active ingredients from PN taproots [6–12]. To the best of our knowledge, no studies have yet investigated the cause of morphological differences in PN taproots, and the metabolic regulation pathways involved in taproot morphology differences remain unknown. Plant traits are shaped by two vital factors, external environment condition and genetic regulation. To a large extent, visible changes in plants are caused by a series of invisible physiological changes and molecular regulation based on transcriptional expression levels, which reveal that gene expression changes are closely correlated with wide variations in plant development characteristics [13]. In recent years, many researchers have sought to understand the regulation mechanism of plant root anatomy and architecture by using the model plant *Arabidopsis thaliana* and the diverse metabolic pathways involved in root development regulation [14]. Consistent with the growth of plant aerial parts, the formation and development of roots follow the internal laws of gene regulation [15]. In other words, changes in root morphology are inevitably accompanied by changes in the internal metabolic pathways. Therefore, research based on transcriptional regulation reveals the causes of plant morphological changes from the perspective of molecular mechanisms. Moreover, the rapid advances in next-generation sequencing (NGS) can provide abundant transcriptome data, which offers insights into the molecular regulation mechanism of plant growth and development, such as rhizome formation in *Nelumbo nucifera*, taproot thickening in PN, fruit morphology of pumpkin cultivars, secondary metabolite accumulation in tuberous roots of *Aconitum heterophyllum*, and improvement of disease resistance in strawberries [16–20]. Collectively, analysis of these data can reveal the internal molecular interaction mechanisms of various plant types, including morphology, yield, and disease resistance.

Both reactive oxygen species (ROS) and class III peroxidase (PRX) play pivotal roles in modulating plant root development. Either ROS balance or PRX activity affects plant root growth and elongation, and this pathway is completely independent of the signaling pathways of plant hormones, such as cytokinin and auxin; this has long been confirmed in *A. thaliana* [21–23]. Additionally, numerous studies have confirmed that ROS are important plant growth regulators and widely involved in various processes of plant root development, such as meristematic expansion and root elongation [24–31]. ROS homeostasis maintains a delicate balance under the coordination of ROS production and scavenging, maintaining an appropriate threshold boundary between redox potential and cytotoxicity [32]. Two important enzymatic families, PRX and NADPH oxidase family, contribute to ROS production [26, 33]. ROS scavenging is a two-armed regulation mechanism. One arm comprises antioxidative enzymes, including catalase (CAT), L-Ascorbate peroxidase (APX), PRX, superoxide dismutase (SOD), and glutathione peroxidase (GPx) [34–37]. The other arm consists of nonenzymatic antioxidants, such as reduced glutathione, ascorbic acid, and flavonoids [38]. It has already been reported that oxidative stress in plant cells is regulated by the mitogen-activated protein kinase (MAPK) family cascade [39]. The MAPK family widely participates in biological processes in plants, such as cell proliferation and differentiation, as well as responding to and tolerating diverse stresses and environmental stimuli [39–41].

Therefore, to investigate the causes of phenotypic differences between LPN and OPN at the molecular regulation level, we performed an in-depth study of the transcriptome data of PN taproot and then verified the results. Three genes were very likely involved in regulating the taproot morphogenesis of PN, which provides a reliable explanation for the phenotypic variation of PN taproot.

## Materials and methods

### Plant materials

PN (three years old) was collected at the vigorous growth stage from three sampling sites: Shiping (SP) county (N23°73′, E103°48′), Shilin (SL) county (N24°77′, E103°27′), and Luxi (LX) county (N24°52′, E103°76′), Yunnan Province, People’s Republic of China. The specimens undertook the formal identification by Xiuming Cui that have been preserved in the greenhouse of the Faculty of Life Science and Technology, Kunming University of Science and Technology. Taproots with obviously different phenotypes (LPN and OPN) were collected separately from individual plants and washed, followed by measurement of their length and width. The samples were immediately stored in liquid nitrogen for further processing. Ten samples were collected from each sampling site, including five LPN and five OPN samples; thus, a total of 30 samples were collected from three sampling sites.

### RNA isolation, library construction, and sequencing

Five samples each of LPN and OPN were collected from each sampling site, for a total of three mixed samples each of LPN and OPN (marked as LPN1, LPN2, and LPN3 for LPN and as OPN1, OPN2, and OPN3 for OPN) for RNA extraction and further experimentation. Total RNA per mixed sample was extracted from the taproot tissue using the RNeasy Plus Mini kit (Qiagen, Valencia, CA, USA), and purified using oligo (dT) magnetic beads. After completing both cDNA library synthesis and PCR amplification for each mixed sample, the PCR product was purified on an AMPure XP system (Beckman Coulter, Beverly, MA, USA). RNA integrity was assessed using an Agilent 2100 Bioanalyzer Instrument (Agilent Technologies, Santa Clara, CA, USA), and RNA degradation and contamination were monitored on 1% agarose gels.

One cDNA library was constructed from each mixed sample, and a total of six libraries were generated. Clustering of the index-coded samples was performed using TruSeq PE Cluster Kit v3-cBot-HS (Illumina, San Diego, CA, USA) based on the cBot Cluster Generation System, following the manufacturer’s instructions. After cluster generation, 150 bp paired-end sequencing were performed using NGS based on the HiSeq 2500 system (Illumina). The clean reads were generated after removing the adapter, ploy-N, and low-quality of raw reads by using fastp software v0.19.4 [42].

### Quality control and de novo assembly

The quality of clean data was evaluated using FASTQC software v0.11.9 (https://www.bioinformatics.babraham.ac.uk/projects/fastqc/). Sequenced reads with a per base average quality score below 28 were filtered, and the first 15 bases of each read were removed using Trimmomatic software v0.39 [43]. To create a better reference unigenes catalog, the LPN1 sample was used as the test object, and then three assemblers including Trinity v2.8.4 [44], SPAdes v3.14.1 [45], and SOAPdenove-trans v1.03 [46] were used to perform *de novo* transcriptome assembly of the LPN1 dataset. At the same time, BUSCO v5.1.0 [47] was used to assess the assembly quality of the three assemblers, as an indication of selecting an optimal assembler. Subsequently, based on the BUSCO assessment result and previous published reports on the comparative analysis of *de novo* transcriptome assemblers [48, 49], we combined all trimmed reads into one sample using Trinity v2.8.4 [44] to complete the assembly process of the reference transcriptome of PN taproot for further analysis. Then, the assembled transcriptome was clustered, and redundancy was removed using CD-HIT software v4.8.1 [50] with a similarity parameter of 0.95. The longest transcripts were extracted as a reference transcriptome for subsequent functional annotation and DEG analysis between LPN and OPN.

### Functional annotation and analysis

Trinotate pipeline v3.2.0 [44] was used to annotate both the reference transcriptome and protein-coding genes predicted by TransDecoder v5.5.0. Trinotate pipeline can integrate several databases including SwissProt, RNAmmer v1.2 (predicting ribosomal RNA), SignalP v5.0 (predicting signal peptides), TMHMM v2.0 (predicting transmembrane helices), and HMMER v3.3.1 (identifying protein domains) to populate its own database. In addition to the databases mentioned above, Trinotate can map BLAST results of the unigenes to GO and KEGG databases to obtain corresponding GO and KO numbers, respectively [51–53]. In brief, Trinotate merged SwissProt, Pfam, and other related databases into the SQLite database. BLASTX with an E-value cut-off of 1.0 × 10^−5^, BLASTP, RNAmmer, SignalP, TMHMM, HMMER, eggNOG, KEGG, and GO homology searches were performed against the SQLite database. Finally, the search results were compiled in a report file as a table. The subcategories of GO terms of the annotated unigenes were visualized using Panther GO-slim, and the related metabolic pathways found from KEGG annotation were visualized using KEGG Mapper [52, 54].

### DEG analysis

The gene expression level of each sample from LPN and OPN was calculated by mapping clean reads back to the reference transcriptome using RNA-seq by expectation-maximization (RSEM) software v1.3.3 [55] and Bowtie 2 v2.4.1 alignment [56]. The expression matrix generated by RSEM was imported into DESeq2 for DEG analysis between LPN and OPN [57]. Genes with adjusted *p* < 0.05 and |log_2_ fold change| > 1 were selected as significant DEGs. The PPI network diagram based on DEGs was constructed using the STRING database (https://string-db.org) with default parameters, and the PPI network was further optimized using Cytoscape software v3.8.2 [58].

### Measurement of H_2_O_2_ content and enzyme activities

The H_2_O_2_ content in OPN and LPN was determined using the H_2_O_2_ content assay kit (Solarbio, Beijing, People’s Republic of China) following the manufacturer’s instructions. The outcome was expressed as H_2_O_2_ content per gram of fresh taproot weight (μmol/g fresh weight). The enzyme activities of APX and PRX in OPN and LPN were measured using APX and PRX assay kits (Solarbio) following the manufacturer's instructions. The enzyme activity was calculated in terms of enzyme activity units per g of fresh taproot weight (U/g fresh weight). Three biological replicates were used for each experiment.

### RT-qPCR validation of target genes

Total RNA was extracted from six PN taproot samples using the TRIzol Reagent (Thermo Fisher Scientific, Waltham, MA, USA). cDNA was reverse-transcribed using the RT6 cDNA Synthesis Kit v2 (Beijing Qingke Biotechnology, Beijing, People’s Republic of China). Specific primers for each gene were designed for RT-qPCR amplification using Primer-BLAST (https://www.ncbi.nlm.nih.gov/tools/primer-blast/). Actin was used as an internal reference gene to normalize the mRNA expression levels of target genes in each sample [59]. The specific primers for the target genes and actin are listed in Supplementary Table 1. Quantitative reactions were performed using the RT-qPCR Detection System (FQD-96A, Hangzhou Bioer Technology, Hangzhou, People’s Republic of China). The reaction mixture (20 μL) contained 10 μL 2× T5 Fast qPCR Mix (SYBR Green I), 0.8 μL each of the forward and reverse primers, and 1 μL of template cDNA. Finally, qPCR amplification was conducted under the following conditions: 95 °C for 60 s, followed by 40 cycles of 95 °C for 15 s, 60 °C for 15 s, and 72 °C for 30 s. The relative expression of internal reference and target genes was calculated using the 2^−ΔΔCT^ method. Three biological replicates were used for the validation study.

## Results

### Statistics of phenotype data

**Fig. 1:**
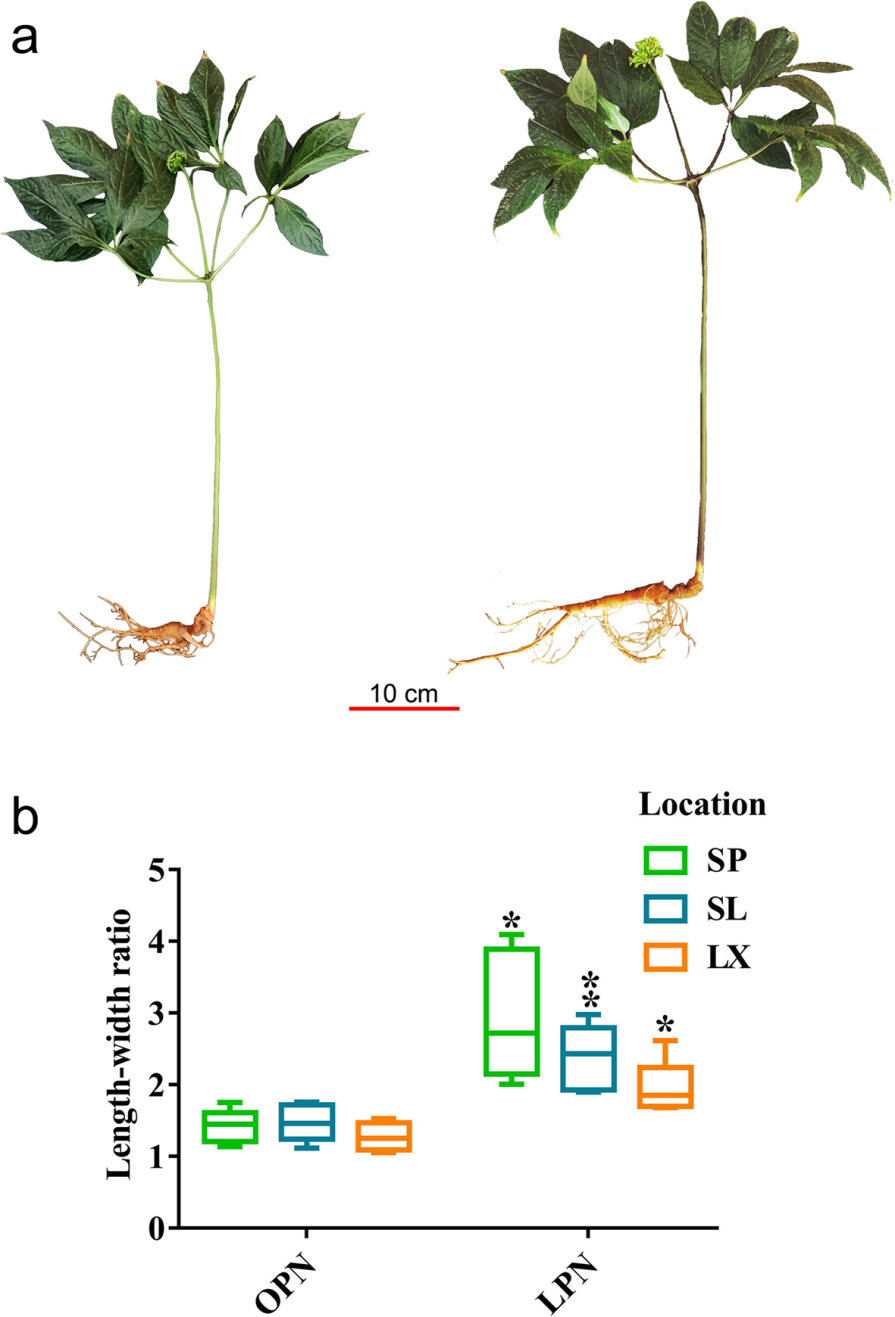
Phenotype characteristics of OPN and LPN taproots**. a** The typical phenotypes of OPN (left) and LPN taproot (right), scale bar: 10 cm. **b** The statistical result of length–width ratios of OPN and LPN taproots. LX, SL, and SP represent Luxi county, Shilin county, and Shipin county, respectively. Data are presented as mean ± s.d. (*n* = 5). The asterisk and double asterisks represent significant difference determined by the independent-sample t-test in PASW at *p* < 0.05 (*) and p < 0.01 (**), respectively.

The typical phenotypes of OPN and LPN are shown in Fig. 1a. Length–width ratios of 30 PN taproot samples (OPN and LPN) collected from Shipin (SP), Shilin (SL), and Luxi (LX) county were calculated. Length–width ratios for OPN and LPN samples collected from the SP county were 1.42 ± 0.23 and 2.96 ± 0.90, those from SL county were 1.47 ± 0.26 and 2.37 ± 0.45, and those from LX county were 1.27 ± 0.20 and 1.95 ± 0.38, respectively. From the calculated results, the length–width ratios of OPN samples were approximately 1.00 to 1.50, whereas those of the LPN samples were approximately 2.00 to 3.00; thus, the length–width ratio of LPN was approximately twice that of OPN. These data were statistically analyzed using the PASW software v18.0.0. Statistical results (Fig. 1b) indicated that there was a significant difference in the length–width ratio between OPN and LPN taproots for each sampling site (*p* < 0.05).

### Illumina sequencing results

After removing adapter, ploy-N, and low-quality reads, Illumina sequencing produced a total of 133,730,886 (39.11 Gb) and 114,761,595 (33.31 Gb) paired-end clean reads from three samples each of OPN and LPN, respectively (Table 1). After assessing read quality using FASTQC software and trimming the first 15 bases of each read using Trimmomatic software, the length of the retained reads was 135 bp, and all of them had a high-quality score (> 28) for subsequent de novo assembly.

**Table 1:** Summary statistics of sequencing data from OPN and LPN using the Illumina HiSeq 2500 system

Sample number	Sample location	Number of total reads	Clean bases (Gb)	Q20 (%)	Q30 (%)
OPN1	SP (N23°73′, E103°48′)	85,408,766	12.52 Gb	97.90	93.91
OPN2	SL (N24°77′, E103°27′)	86,151,720	12.54 Gb	98.52	95.40
OPN3	LX (N24°52′, E103°76′)	95,901,286	14.05 Gb	98.00	94.17
LPN1	SP (N23°73′, E103°48′)	72,558,374	10.64 Gb	98.09	94.32
LPN2	SL (N24°77′, E103°27′)	78,429,508	11.49 Gb	97.47	92.88
LPN3	LX (N24°52′, E103°76′)	78,535,308	11.18 Gb	97.61	93.26

*Abbreviations: OPN* oval taproot of *Panax notoginseng* (Burk.) F. H. Chen, *LPN* long taproot of *Panax notoginseng* (Burk.) F. H. Chen, *SP* Shipin county, *SL* Shilin county, *LX* Luxi county

### *De novo* transcriptome assembly and function annotation

**Fig. 2:**
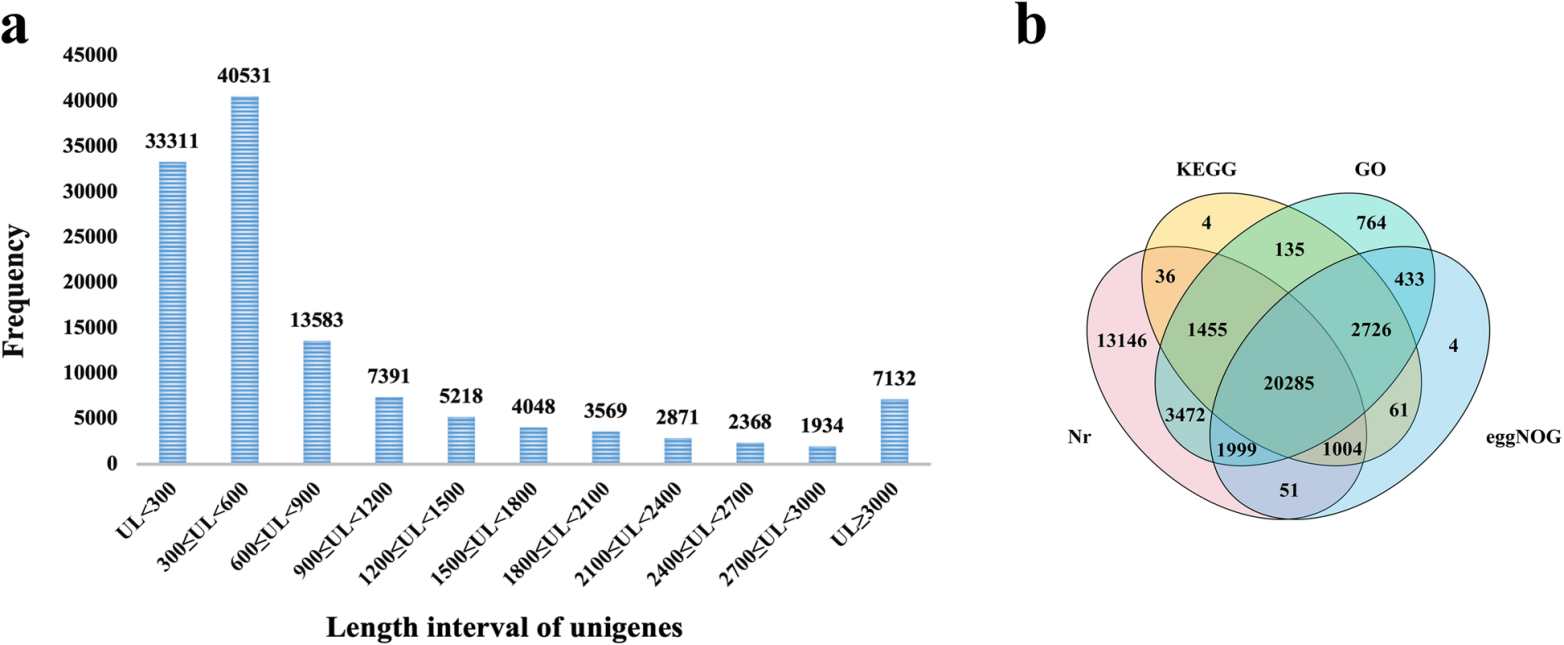
Statistical results of length distribution and database annotation of the reference transcriptome. **a** The statistical results of unigenes length (UL) distribution. **b** The annotation result statistics for all unigenes annotated to the four databases, *namely Nr, KEGG, GO and eggNOG*

BUSCO assessment results based on test sample dataset (LPN1) suggested that the percent of complete BUSCOs (n: 1614) of Trinity, SPAdes and SOAPdenove-trans is 81.84, 73.54 and 68.09%, respectively. Additionally, the transcriptome of LPN1 assembled by Trintiy, SPAdes, and SOAPdenove-trans consisted of 156,549 transcripts (contig N50 = 1,634 bp), 143,719 transcripts (contig N50 = 1,512 bp), and 177,819 transcripts (with contig N50 = 1,121 bp), respectively. The assessment results shown that Trinity is superior to the other assemblers for PN transcriptome assembly (Supplementary Table 2). The reference transcriptome generated from Trinity (after clustering, moving redundancy, and extracting longest transcripts) consisted of 121,955 unigenes with an average length of 914 bp, contig N50 = 1,774 bp, and GC content percentage = 38.27%; the length distribution statistics of all unigenes are shown in Fig. 2a.

In total, 42,207 protein-coding genes were predicted from the reference transcriptome using the TransDecoder software. The final annotation results showed 38,313 Basic Local Alignment Search Tool (BLASTX) hits and 24,820 BLASTP hits. Moreover, 41,448, 25,706, 31,269, and 26,563 unigenes were mapped to RefSeq non-redundant proteins (Nr), the Kyoto Encyclopedia of Genes and Genomes (KEGG), Gene Ontology (GO), and eggNOG databases, respectively, and 20,285 unigenes were common among the Nr, KEGG, GO, and eggNOG databases (Fig. 2b).

**Fig. 3:**
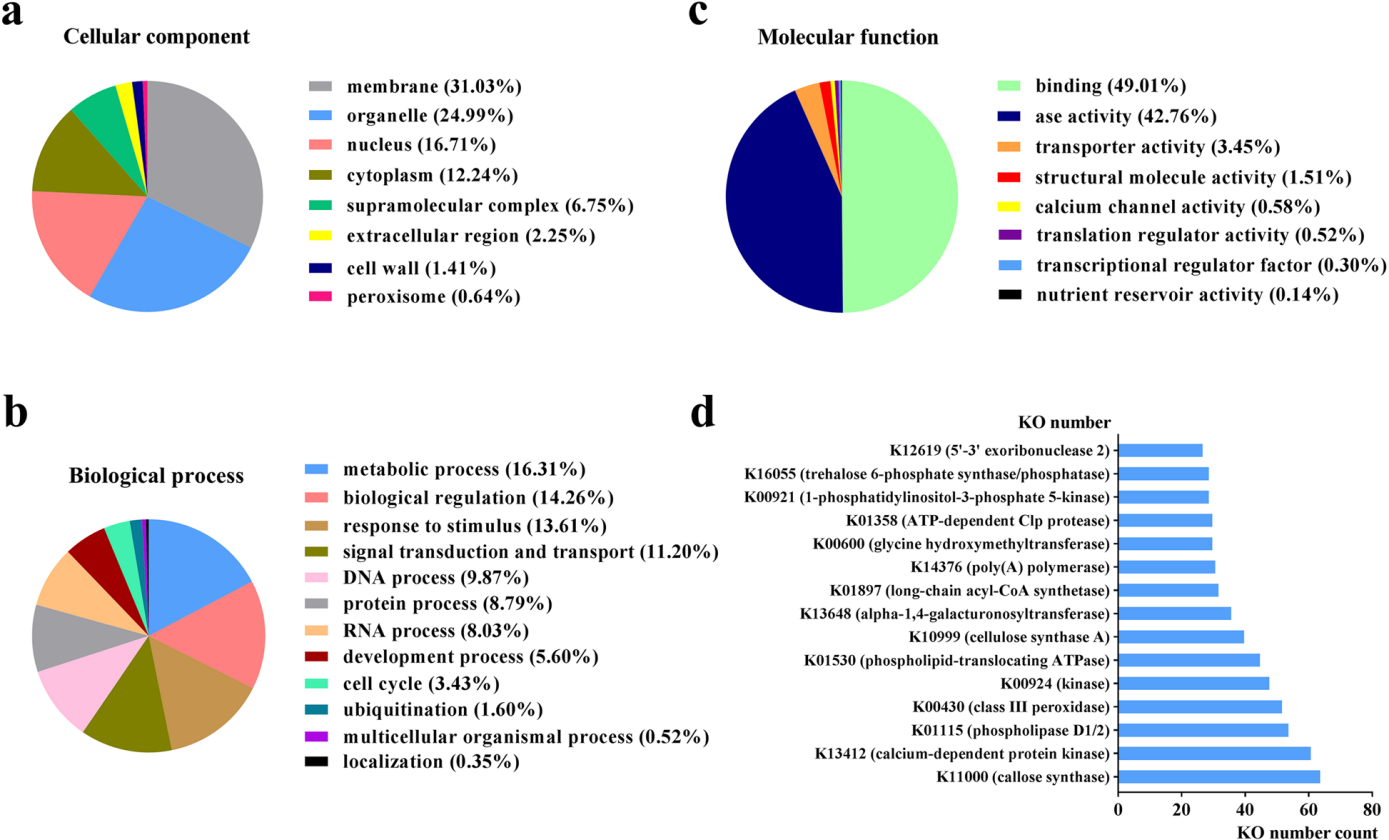
The GO classification results of annotated unigenes and the top 15 KO numbers. Pie charts indicate the percentage of genes classified into diverse GO-slim terms for **a** cellular components, **b** biological processes, and **c** molecular functions. **d** the statistics of the top 15 KEGG Orthology (KO) numbers mapped to the KEGG database

GO function classification distinguished the annotated unigenes involved in different cellular components, biological processes, and molecular functions, and then divided them into subcategories within each GO term domain. Within the cellular component subcategory (Fig. 3a), the majority of annotated unigenes were in the membrane (31.03%), organelle (24.99%), and nucleus (16.71%) subcategories, and others were distributed in the cytoplasm (12.24%), supramolecular complex (6.75%), extracellular region (2.25%), cell wall (1.41%), and peroxisome (0.64%) subcategories. Within the biological process subcategories (Fig. 3b), a high percentage of annotated unigenes were involved in metabolic processes (16.31%), biological regulation (14.26%), response to stimulus (13.61%), and signal transduction and transport (11.2%). The rest were mainly involved in RNA processing (8.03%), DNA processing (9.87%), protein processing (8.79%), development process (5.60%), and cell cycle (3.43%). Within the molecular function subcategories (Fig. 3c), 49.01% of annotated unigenes were involved in binding, 42.76% were involved in enzyme activity, 3.45% were involved in transporter activity, 1.51% participated in structural molecule activity, 0.58% participated in calcium channel activity, and 0.52% participated in translation regulator activity.

### Mapping of KEGG pathways

The analysis of metabolic pathway assignment from the annotated unigenes was performed using the KEGG database. The KEGG annotation results showed that 16,225 unigenes were assigned to specific metabolic pathways with corresponding KEGG Orthology (KO) numbers; among them, 10,575 unigenes matched KO numbers in the KEGG database for *A. thaliana* and are involved in various metabolic pathways. Then, the repeated KO numbers were added up and counted. The top 15 KO numbers (with corresponding definition) mapped to enzymes are shown in Fig. 3d. The total number of genes encoding PRX (K00430) ranked fourth in PN reference transcriptome, which indicates that *PnPRX* may play an extremely important role in the PN taproot development processes. Moreover, all known enzymes involved in the metabolism pathway of the ROS and MAPK families were identified in the KEGG database for *A. thaliana*. Among the genes encoding enzymes related to ROS metabolism, 42 genes belong to PRX gene family, 16 genes belong to GPx gene family, and 8 genes belong to APX gene family were identified. In addition, KEGG analysis identified 55 genes belong MAPK gene family related to plant growth, development, and response to oxidative stress, of which 73 were mapped to the KEGG database for *A. thaliana*, and 21 of the 73 genes were assigned to specific metabolic pathways with corresponding KO numbers.

### Analysis of differentially expressed genes (DEGs)

**Fig. 4:**
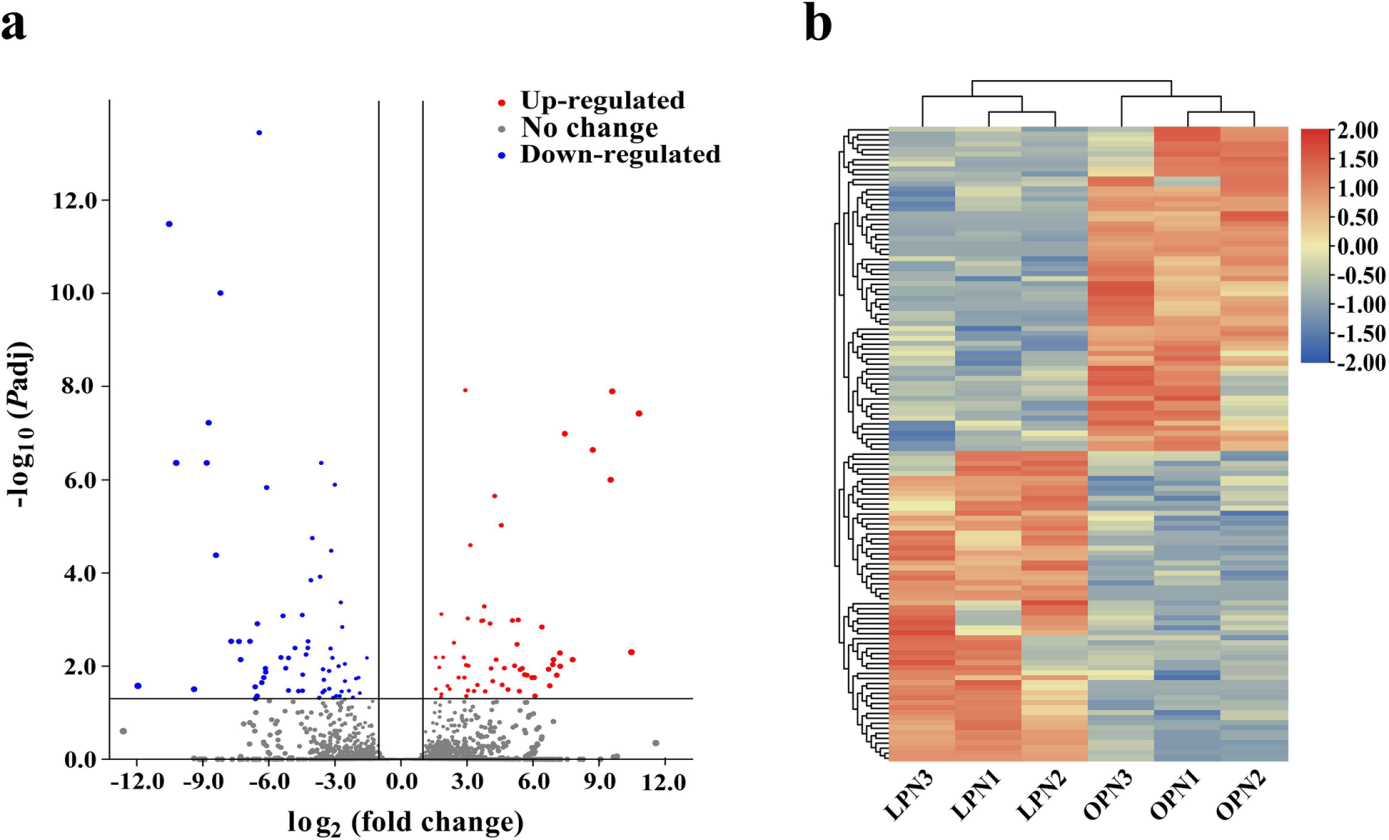
Volcano plot of gene expression levels and heatmap of differentially expressed genes (DEGs). **a** The volcano plot was generated from gene-level differential expression results based on DESeq2, showing the distribution of down- and up-regulated genes in LPN as compared with in OPN. DESeq2 showed 65 down-regulated and 62 up-regulated DEGs in LPN. In blue are the significantly (*P*_adj_ < 0.05) down-regulated genes with a log_2_ fold change (LFC) < −1 (left of the vertical line). In red are the significantly (*P*_adj_ < 0.05) up-regulated genes with an LFC > 1 (right of the vertical line). The larger the absolute value of LFC, the larger the area of the point. **b** Clustering heatmap of 127 DEGs. The heatmap shows down-regulated and up-regulated genes between LPN (LPN1, LPN2 and LPN3) and OPN (OPN1, OPN2 and OPN3) samples (LFC < −1 or > 1, *P*_adj_ < 0.05). Color intensity represents expression level of DEGs, the deeper the red, the higher the gene expression level in samples, whereas the deeper the blue, the lower the expression in samples

The volcano plot of gene expression levels illustrates the distribution of up- and down-regulated genes in LPN as compared with in OPN (Fig. 4a). A total of 127 DEGs were identified between LPN and OPN, and the heatmap clearly presents the clustering results of these DEGs in OPN and LPN samples, showing that genes that were significantly up-regulated in LPN were significantly down-regulated in OPN (Fig. 4b). Of the identified 127 DEGs, 83 were successfully annotated with BLASTX hits. Among the 83 annotated genes, 44 were up-regulated and 39 were down-regulated in LPN taproot, and 27 genes (Supplementary Table 3) including 18 up-regulated and 9 down-regulated genes were assigned to specific metabolic pathways with corresponding KO numbers. Among the 18 up-regulated genes, the three genes are very likely to be related to the phenotypic difference between LPN and OPN, including two genes encoding enzymes originating from APX ([EC: 1.11.1.11]) and PRX ([EC: 1.11.1.7]), respectively, both enzymes are directly related to ROS metabolism, and one gene encoding an enzyme of the MAPK [EC: 2.7.11.24] related to plant cell proliferation and meristematic maintenance (Table 2). Specifically, the *PnAPX3*, *PnPRX45* and *PnMPK3* genes, respectively, according to the results of BLASTP hit. In total, 47 DEGs were integrated into the protein–protein interaction (PPI) network diagram (Supplementary Figure 1), including 30 up-regulated (purple nodes) and 17 down-regulated DEGs (green nodes), with five genes involved in ATP binding. Moreover, PPI networks illustrate that both *PnAPX3* and *PnMPK3* directly interact with *PnRCA* gene, as shown by the two straight lines among the three purple nodes in a red circle (Supplementary Figure 1). GO annotation results showed that the molecular function of gene *PnRCA* was implicated in ATP binding. This suggested that *PnAPX3* and *PnMPK3* may be indirectly involved in the energy metabolism of PN root cells.

**Table 2:** Pathway assignments of enzymes encoded by the three up-regulated genes in LPN

Enzyme	Definition	Gene name	***A. thaliana*** gene	LFC	***P***adj
[EC: 1.11.1.11]	L-Ascorbate peroxidase	*PnAPX3*	AT4G35000	4.56	9.42E-06
[EC: 1.11.1.7]	Class III peroxidase	*PnPRX45*	AT4G30170	3.73	0.001047
[EC: 2.7.11.24]	Mitogen-activated protein kinase	*PnMPK3*	AT3G45640	2.92	1.21E-08

*Abbreviations*: *LFC* log_2_ (fold change), *Padj* adjusted *P* value

### Measurement results of H_2_O_2_ content and enzyme activity

**Fig. 5:**
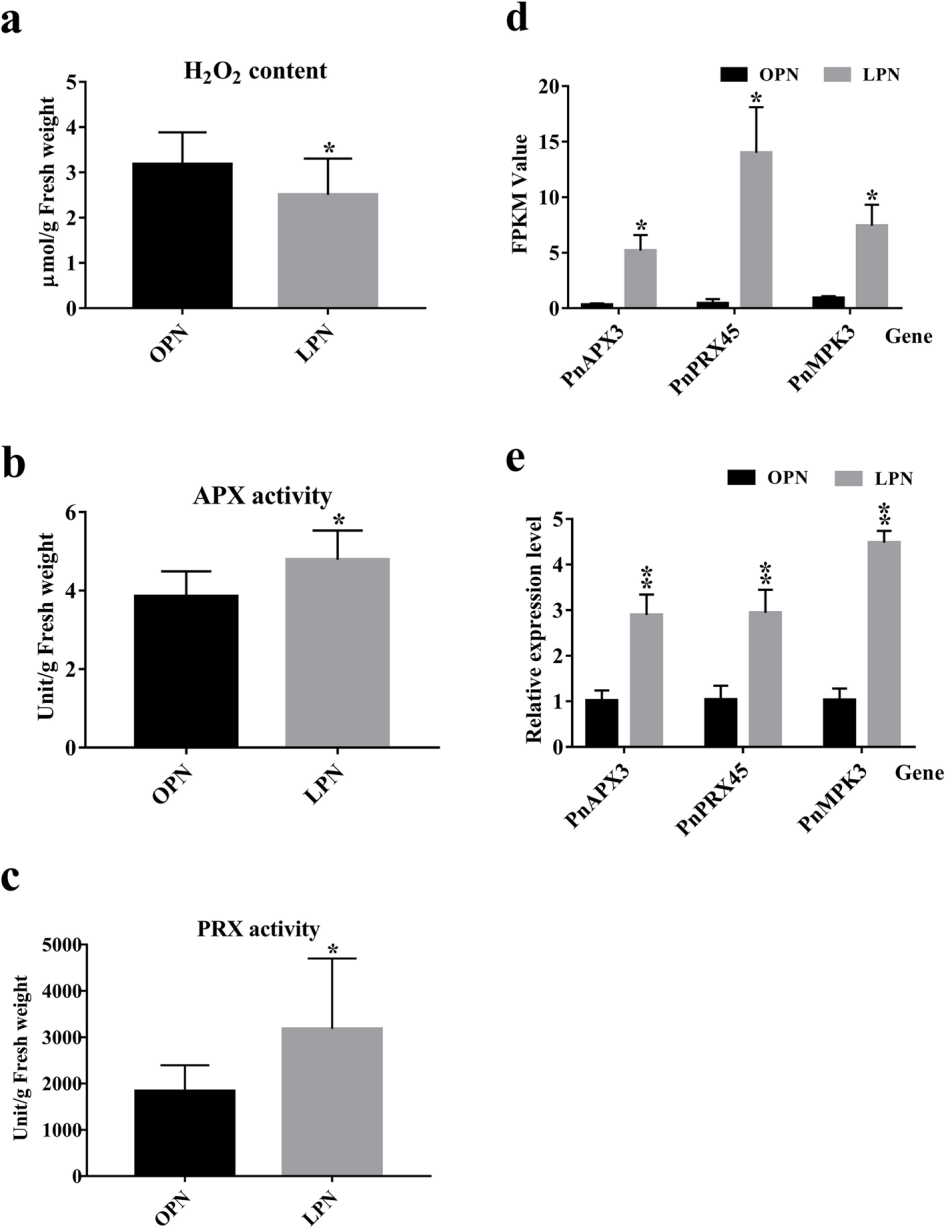
The determination results of H_2_O_2_, APX, PRX, RT-qPCR, and the statistics of FPKM. **a** H_2_O_2_ concentration and enzyme activities of **b** APX and **c** PRX, as well as **d** statistics of fragments per kilobase million (FPKM) value of the three target genes (*PnAPX3*, *PnPRX45* and *PnMPK3*), and **e** RT-qPCR validation results. Data are presented as mean ± s.d. (*n* = 3). The asterisk and double asterisks represent significant differences determined by the independent-sample t-test in PASW at *p* < 0.05 (*) and *p* < 0.01 (**), respectively

APX and PRX enzyme activities and H_2_O_2_ concentrations were measured in OPN and LPN. The H_2_O_2_ concentrations in OPN and LPN were 3.18 ± 0.70 and 2.50 ± 0.80 (μmol/g fresh weight), respectively (Fig. 5a). The statistical results showed that there was a significant difference in the H_2_O_2_ content between OPN and LPN (*p* < 0.05). The enzyme activities of APX and PRX in OPN were 3.85 ± 0.64 and 1837.08 ± 556.74 (U/g fresh weight), and those in LPN taproots were 4.79 ± 0.75, 3177.54 ± 1522.90 (U/g fresh weight), respectively. The statistical results showed that the activities of the two antioxidant enzymes were significantly up-regulated in LPN compared with in OPN (*p* < 0.05) (Fig. 5b and c). Thus, up-regulated APX and PRX enzyme activities led to lower H_2_O_2_ content in LPN taproots.

### RT-qPCR analysis results

The fragments per kilobase million (FPKM) values of the *PnAPX3*, *PnPRX45*, and *PnMPK3* in OPN were 0.31 ± 0.13, 0.44 ± 0.37, and 0.93 ± 0.16, whereas those in LPN were 7.43 ± 1.89, 5.20 ± 1.40, and 14.00 ± 4.10, respectively. The statistical results of FPKM values showed that the relative expression levels of the *PnAPX3*, *PnPRX45*, and *PnMPK3* in LPN were significantly higher than in OPN (*p* < 0.05) (Fig. 5d). The relative expression levels of the three genes in OPN calculated based on RT-qPCR were 1.03 ± 0.31, 1.01 ± 0.22, and 1.03 ± 0.25, whereas those in LPN were 2.89 ± 0.45, 4.48 ± 0.26 and 2.94 ± 0.50, respectively. Interestingly, the relative expression levels of the three genes in LPN were approximately 3-, 4-, and 3-fold higher than those in OPN, respectively. Thus, the overall relative expression levels of *PnAPX3*, *PnPRX45*, and *PnMPK3* genes were significantly higher in LPN than in OPN (*p* < 0.01) (Fig. 5e).

Both RT-qPCR and statistical analyses results of FPKM values confirmed the analysis of DEGs; that is, the relative expression levels of the *PnMPK3*, *PnAPX3*, and *PnPRX45* genes were up-regulated in LPN.

## Discussion

ROS are extremely active chemical substances and mainly exist in two forms: radicals with free electrons such as superoxide (O_2_^-^) and hydroxyl radical (OH•) and nonradicals such as hypochlorous acid (HOCl) and hydrogen peroxide (H_2_O_2_) [60]. Different types of ROS have different chemical properties; H_2_O_2_ is the most stable ROS molecule [61, 62]. Depending on the type and concentration of ROS produced in the cells, different physiological reactions may occur. ROS can induce the expression of stress-responsive genes at low concentrations but can lead to oxidative damage to important biomacromolecules such as lipids, nucleic acids, and proteins, eventually causing cell death at high concentrations [63, 64]. To date, many studies have reported important roles of ROS, including regulation of growth throughout plant root development, maintenance of apical meristem, and promotion of root elongation; the related internal molecular mechanisms have also been explored [24]. Specifically, the molecular mechanism of ROS-mediated control of the cell cycle, including cellular proliferation, elongation, and differentiation, has been widely demonstrated in *A. thaliana* and other plants [25–30, 60].

**Fig. 6:**
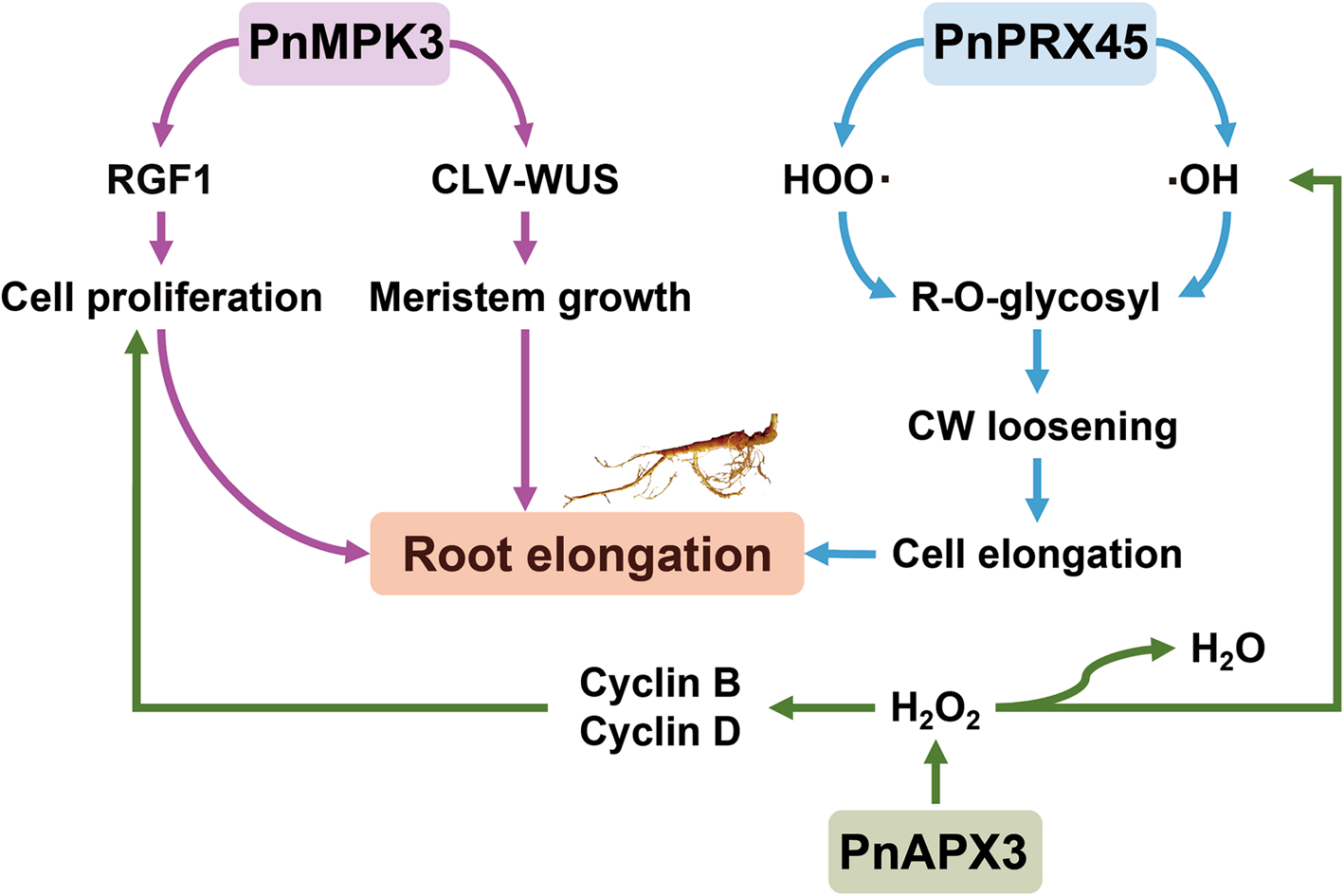
A Putative network diagram of molecular regulatory mechanism leading to LPN formation. The blue, green, and purple arrows represent the metabolic pathways involving *PnPRX45*, *PnAPX3* and *PnMPK3*, respectively

Plant cell expansion and elongation are mostly determined by the plasticity and structure of the cell wall (CW). The cellular expansion rate, namely cellular dynamics, is closely associated with the balance between CW stiffening and loosening, both of which are controlled by ROS metabolic processes [26]. Thus, ROS production and scavenging are directly related to the elongation and development of plant roots. ROS is generated mainly by the PRX and NADPH oxidase families [26, 33], whereas its scavenging is mainly regulated by antioxidant enzymes such as CAT, APX, PRX, SOD, and some nonenzymatic antioxidants, such as reduced glutathione and ascorbic acid [34, 38]. A number of early studies on *A. thaliana* have confirmed that accumulation of H_2_O_2_ in plant root cells can enhance CW rigidity to hinder cellular elongation and proliferation by repressing the expression of cell cycle-related genes *CyclinB* and *CyclinD*, thus leading to shorter roots. In contrast, scavenging an excess of H_2_O_2_ in plant root cells contributes to maintenance of intracellular redox homeostasis in favor of cellular proliferation rather than differentiation, thus leading to the development of longer roots [21, 23, 26, 28]. In the present study, it was found that the H_2_O_2_ concentration in LPN was significantly lower than that in OPN (*p* < 0.05) (Fig. 5a). As shown by green arrows in Fig. 6, low H_2_O_2_ concentration in root cell, which is conducive to the expression of cell cycle-related genes *CyclinB* and *CyclinD*, to promote cell division and root growth. Therefore, it is possible that the emergence of the LPN phenotype is tightly correlated with lower H_2_O_2_ concentration caused by the high expression of some antienzymes in root cells.

APXs, bifunctional enzymes with CAT and broad-spectrum peroxidase activity, are the core enzymes that scavenge ROS in plants [65, 66]. In plant cells, APXs scavenge H_2_O_2_, which mainly participates in the first step of the ascorbate-glutathione cycle, catalyzing the reaction of L-ascorbate + H_2_O_2_ + 2 H^+^ = L-ascorbate + L-dehydroascorbate + 2 H_2_O to convert excess H_2_O_2_ into H_2_O [67]. During plant development, an increasing number of APX-encoding genes, such as APX1–6, were detected in *A. thaliana*, and their activity and function have been further confirmed [68]. In addition, other APX-encoding genes have been found in rice, wheat, and potato tubers. A number of studies on the effects of APX-knockout genes on plant physiological functions, growth processes, and antioxidant metabolism suggest that APXs can influence plant growth and development by regulating cellular redox signaling pathways involved in plant growth [69–73]. The up-regulated expression of gene *PnAPX3* in LPN was validated by RT-qPCR results (Fig. 5e) and APX enzyme activity analysis (Fig. 5b) in this study. Based on the known function of APX3, up-regulated *PnAPX3* also confirmed the lower content of H_2_O_2_ in LPN compared with in OPN. Therefore, it is reasonable to infer that the up-regulated *PnAPX3* in LPN can maintain the content of H_2_O_2_ in cells at a lower level compared with that in OPN, increase the rate of cell proliferation, and promote root elongation (green arrows in Fig. 6)

PRXs are plant CW-localized proteins that have been shown to be involved in plant CW dynamics [74]. PRXs can generate ROS such as •OH and HOO• to promote cell elongation in the hydroxylic cycle and oxidize various substrates to polymerize typical CW lignin compounds to stiffen the CW in the peroxidative cycle; both cycles are capable of regulating the H_2_O_2_ level [26, 75]. To be specific, during the peroxidative cycle, some PRXs can oxidize molecules such as monolignols, suberin units, and ferulic acids linked to diverse polymers to form impregnable cross-links between CW polymers and proteins in CW network, leading to a tight CW, which directly reduces cellular elongation and expansion capacity [23, 76]. However, during the hydroxylic cycle, other PRXs with extremely strong activity can produce •OH and HOO•, which can nonspecifically break polysaccharide covalent bonds in various types of organic molecules to enhance nonenzymatic CW loosening and consequently promote cellular elongation [26]. A previous study on *A. thaliana* confirmed that the expression inhibition of genes encoding PRXs results in an increase in H_2_O_2_ content, whereas up-regulated expression of genes encoding PRXs can continually scavenge excess H_2_O_2_ in plant root cells and maintain intracellular redox homeostasis in favor of cellular proliferation rather than differentiation, leading to the development of longer roots [21]. Briefly, the function of PRXs can be classified into two main categories: stiffening and loosening CW. Additionally, based on previous studies of multiple phenotypes of the corresponding AtPrx-deficient mutants of *A. thaliana* (such as mutants deficient in *AtPrx02/25/71*, *AtPrx33/34*, and *AtPrx53*), one PRX category, including *AtPrx37* [77], *AtPrx02/25/71* [78], *AtPrx64* [79], and *AtPrx72* [80], can promote the hardening of the plant CW by taking H_2_O_2_ as an electron acceptor to catalyze lignin formation and polymerization [81–83]. In other words, they can catalyze lignin formation and polymerization to stiffen the plant CW in the presence of H_2_O_2_, thus leading to shorter roots. Other categories of PRXs, including *AtPrx36*, *AtPrx39*, *AtPrx40*, *AtPrx57*, *AtPrx33/34*, and *AtPrx53*, were found to be closely associated with CW loosening and root elongation [22, 84, 85]. Specifically, *A. thaliana* with overexpression of gene encoding PRX34 had longer roots than the wild type (WT), whereas the plants with double knockdown of genes encoding PRX33 and PRX34 had shorter roots than the WT. The gene identified in this study, which *PnPRX45* (named *AtPrx45* in *A. thaliana*), was significantly up-regulated in LPN compared with in OPN (Fig. 5c). PRX45, like other PRXs, mainly exists in roots and hypocotyl tissues and acts in response to oxidative stress according to description in *A. thaliana* database. However, the specific molecular regulation mechanism of PRX45 in the plant root CW has not been reported. The up-regulated expression of the gene *PnPRX45* in LPN was validated by RT-qPCR results (Fig. 5e) and PRX enzyme activity analysis (Fig. 5c). It is reasonable to speculate that *PnPRX45* may participate in the hydroxylic cycle to produce •OH and HOO• and split glycosidic bonds or certain covalent bonds in CW components, resulting in cell elongation and expansion, and eventually root elongation (blue arrow in Fig. 6). The specific molecular function of PRX45 may be comprehensively confirmed in *A. thaliana* roots, similar to other AtPrx-encoding genes in the foreseeable future.

MAPKs, also known as MPKs in plants, are a highly conserved enzyme family with essential functions; they are widely found in plant and animal cells. The MAPK cascade is composed of diverse proteinases involved in various biological processes, including cell proliferation, differentiation, response to diverse stresses, and tolerance to environmental stimuli [39–41]. For instance, it has been shown that the activity of *PsMPK2* in pea and *ZmMPK5* in maize can be elevated by H_2_O_2_ stress [86, 87]. *TaMPK4* plays a critical role in mediating plant tolerance to various stresses by inducing root growth and regulating cellular ROS metabolism [88]. *StMAPK11* upregulation can enhance CAT and PRX activity to increase the antioxidant activity in potato, tobacco, and *A. thaliana* [89]. The activities of MPK3 and MPK6 in *A. thaliana* are positively correlated with plant defense against oxidative stress triggered by salt stress [90]. Importantly, the regulatory functions of the MAPK cascade in plant shoot apical meristem (SAM) have long been proposed, as MPK3 and MPK6 are crucial regulators of stem cell homeostasis in *A. thaliana* by participating in CLAVATA peptide receptor-WUSCHEL transcription factor (CLV-WUS) signaling pathways of SAM development [91, 92]. In addition, two recently reported studies indicated that MPK3 can positively regulate root meristem growth factor 1 (*RGF1*)-mediated root growth and development and promote cell division in the root apical meristem, leading to plant root elongation [93, 94]. In the present study, RT-qPCR was conducted to validate the expression of the *PnMPK3* gene in LPN and OPN. The validation results were consistent with the analysis of DEGs; the *PnMPK3* gene was up-regulated in LPN as compared with in OPN (Fig. 5e). Based on recent studies on MPK3 function in *A. thaliana*, it is rational to assume that up-regulated *PnMPK3* gene is more favorable for stem cell maintenance of plant root meristem and can mediate *RGF1* expression, leading to promotion of cell proliferation in the PN taproot. Therefore, up-regulated *PnMPK3* activity is likely to be one of the driving forces for the formation of the LPN phenotype (purple arrows in Fig. 6).

Additionally, previous studies have confirmed that plant root development is jointly determined by the rates of cell proliferation and the extent of cell elongation [30, 95]. Several published studies on different sweet potato root types suggest that up-regulated antioxidant enzyme levels could improve plant root growth and development, and under certain circumstances, may increase yield [26, 33, 96]. In the present study, DEGs analysis showed that the *PnAPX3*, *PnPRX45*, and *PnMPK3* genes were significantly up-regulated in LPN as compared with in OPN. Further, H_2_O_2_ content, APX and PRX enzyme activity, and RT-qPCR analyses in LPN and OPN further verified the transcriptome analysis. This illustrates that *PnAPX3*, *PnPRX45*, and *PnMPK3* may be directly or indirectly involved in the process of promoting PN taproot development and elongation. A hypothetical molecular regulatory network leading to LPN formation may be based on the joint interference of H_2_O_2_, *PnAPX3*, *PnPRX45*, and *PnMPK3* (Fig. 6).

## Conclusions

In this study, we performed *de novo* transcriptome assembly and functional annotation from six PN taproot samples (three each of LPN and OPN) and determined the causes of phenotypic differences in the development process of PN by analyzing DEGs of LPN and OPN. DEGs analysis showed that *PnAPX3*, *PnPRX45*, and *PnMPK3* genes were significantly up-regulated in LPN compared with in OPN. These three enzymes (APX, PRX and MPK) play pivotal roles in ROS metabolism and oxidative stress. Many previous studies have demonstrated that the process of ROS metabolism is closely related to the proliferation, elongation, and differentiation of plant root cells. It has recently been reported that MPK3 can positively regulate RGF1-mediated root growth and is indispensable for stem cell maintenance in the shoot apical stem of *A. thaliana*.

In summary, we confirmed that the PN taproot phenotype is influenced by a network controlling ROS metabolism during the taproot development process. Based on the results of this study and those of previously published studies on the relationship between ROS metabolism and plant root development, it can be concluded that the taproot phenotype of LPN is due to the up-regulated expression of *PnAPX3*, *PnPRX45*, and *PnMPK3*. The results of this study provide a reliable explanation for the phenotypic differences between OPN and LPN, and they offer further insights into the genetic mechanism of phenotypic differences for other species of the *Panax* genus. Our results will be useful for future molecular breeding of PN.

## Supplementary Information

**Additional file 1: Supplementary Figure 1. **The protein-protein interaction (PPI) network diagram based on differentiallyexpressed genes (DEGs). Green represents down-regulated genes and purple represents up-regulated genes.

**Additional file 2:** **Supplementary Table 1.** The specific primers of the three target genes and internal reference gene (actin).

**Additional file 3:** **Supplementary Table 2.** BUSCO assessment the assembly results of test sample dataset (LPN1) based on three assemblers.

**Additional file 4:** **Supplementary Table 3.** Basic information of 27 DEGs mapped to specific metabolic pathways with corresponding KO numbers.

## Data Availability

The datasets supporting the conclusion of this article are included within the article and its additional files, and the sequencing data for all samples is available in the NCBI database with the BioProject number PRJNA782237.
