# Supplementary figures and images for: De novo and comparative transcriptomic analysis explain morphological differences in Panax notoginseng taproots
Source: BMC Genomics. 2022 Jan 31;23:86. doi: 10.1186/s12864-021-08283-w (PMC8802446; doi:10.1186/s12864-021-08283-w)

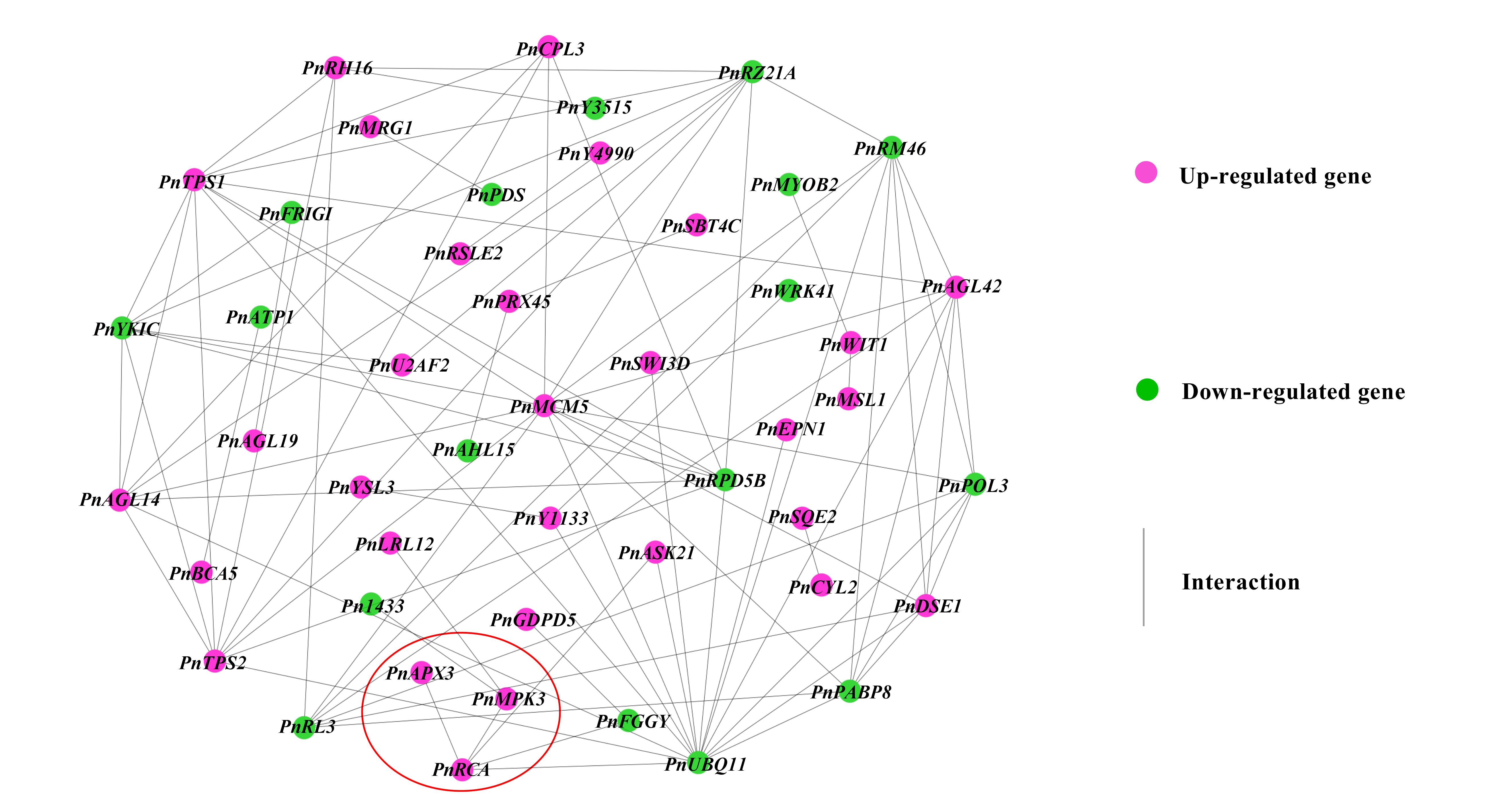

Supplement: Supplementary file 1 — Additional file 1: Supplementary Figure 1. The protein-protein interaction (PPI) network diagram based on differentiallyexpressed genes (DEGs). Green represents down-regulated genes and purple represents up-regulated genes. [file 12864_2021_8283_MOESM1_ESM.jpg]
