# Supplementary material for: De novo and comparative transcriptomic analysis explain morphological differences in Panax notoginseng taproots
Source: BMC Genomics. 2022 Jan 31;23:86. doi: 10.1186/s12864-021-08283-w (PMC8802446; doi:10.1186/s12864-021-08283-w)
Supplement: Supplementary file 2 — Additional file 2: Supplementary Table 1. The specific primers of the three target genes and internal reference gene (actin). [file 12864_2021_8283_MOESM2_ESM.docx]

**Supplementary table 1** The specific primers of the three target genes and internal reference gene (actin).

| **Gene** | **Primer sequences （5’🡪3’）** | **The number of bases** |
| --- | --- | --- |
| Actin | TCCAAGGGTGAATATGATGAATCG-F | 24 |
|  | AACCTCTCCAAAGAGAATTTCTGAGT-R | 26 |
| *PnAPX3* | GTGGTAGACACCGAGTATT-F | 19 |
|  | ATTATTAGAGCCGTGAGAGTAT-R | 22 |
| *PnPRX45* | CGGTTATCAAAGCCAAGGCG-F | 20 |
|  | TCCCATCTCGTCTTCCCAGT-R | 20 |
| *PnMPK3* | ACCGCAACCATCTCATTCGT-F | 20 |
|  | ATACCGGCCTCCGATTATGC-R | 20 |

F represents Forward primer; R represents Reverse primer.
