# Supplementary material for: De novo and comparative transcriptomic analysis explain morphological differences in Panax notoginseng taproots
Source: BMC Genomics. 2022 Jan 31;23:86. doi: 10.1186/s12864-021-08283-w (PMC8802446; doi:10.1186/s12864-021-08283-w)
Supplement: Supplementary file 3 — Additional file 3: Supplementary Table 2. BUSCO assessment the assembly results of test sample dataset (LPN1) based on three assemblers. [file 12864_2021_8283_MOESM3_ESM.docx]

**Supplementary Table 2** BUSCO assessment the assembly results of test sample dataset (LPN1) based on three assemblers.

|  | **Trinity** | **SPAdes** | **SOAPdenovo-trans** |
| --- | --- | --- | --- |
| **BUSCO assessment results (n: 1614)** |  |  |  |
| Complete BUSCOs | 1,321 (81.84%) | 1,187 (73.54%) | 1,099(68.09%) |
| Fragmented BUSCOs | 154 | 244 | 271 |
| Missing BUSCOs | 139 | 183 | 244 |
| **Assembly results** |  |  |  |
| The number of transcripts | 156,549 | 143,179 | 177,819 |
| Contig N50 (bp) | 1,634 | 1,512 | 1,121 |
| Average contig length (bp) | 968 | 812 | 498 |
| Median contig length (bp) | 564 | 396 | 212 |
