# Supplementary material for: De novo and comparative transcriptomic analysis explain morphological differences in Panax notoginseng taproots
Source: BMC Genomics. 2022 Jan 31;23:86. doi: 10.1186/s12864-021-08283-w (PMC8802446; doi:10.1186/s12864-021-08283-w)
Supplement: Supplementary file 4 — Additional file 4: Supplementary Table 3. Basic information of 27 DEGs mapped to specific metabolic pathways with corresponding KO numbers. [file 12864_2021_8283_MOESM4_ESM.docx]

**Supplementary Table 3** Basic information of 27 DEGs mapped to specific metabolic pathways with corresponding KO numbers

| **Results of BLASTX hit** | **Description in Pfam** | **Annotation results of eggNOG database** | **KO number** | **Definition in KEGG database** | **expression** |
| --- | --- | --- | --- | --- | --- |
| *PUX1_ARATH* | Regulator of chromosome condensation repeat | Alveolar soft part sarcoma chromosome region, candidate 1 | K15627 | Tether containing UBX domain for GLUT4 | Up |
| *PLA2A_ARATH* | Phosphotransferase enzyme family | Phospholipase A2 | K01047 | Secretory phospholipase A2 | Up |
| *GDPD5_ARATH* | Glycerophosphoryl diester phosphodiesterase family | Glycerophosphoryl diester phosphodiesterase | K01126 | Glycerophosphoryl Diester phosphodiesterase | Up |
| *MPK3_ARATH* | Protein kinase domain | Mitogen-activated protein kinase | K04371 | Mitogen-activated protein kinase | Up |
| Null | Null | RNA binding motif protein | K19410 | Inner nuclear membrane protein Man1 | Up |
| *PABP8_ARATH* | GTPase domain | Poly(A) binding protein, cytoplasmic | K13126 | Polyadenylate-binding protein | Up |
| *APX3_ARATH* | Peroxidase | Bifunctional enzyme with both catalase and broad- spectrum peroxidase activity | K00434 | L-ascorbate peroxidase | Up |
| *PER45_ARATH* | Peroxidase | Peroxidase | K00430 | Peroxidase | Up |
| *CPL3_ARATH* | GFD1 mRNA transport factor | Carboxy-terminal domain, RNA polymerase II, polypeptide A | K18999 | RNA polymerase II C-terminal domain phosphatase-like | Up |
| *SEC_ARATH* | Tetratricopeptide repeat | Protein N-acetylglucosaminyltransferase activity | K09667 | Protein O-GlcNAc transferase | Up |
| *BCA5_ARATH* | Carbonic anhydrase | Carbonic anhydrase | K01673 | Carbonic anhydrase | Up |
| *SPPA1_ARATH* | Biotin protein ligase C terminal domain | Signal peptide peptidase, SppA | K04773 | protease IV | Up |
| *PSBC_CHLSC* | ABC transporter | (ABC) transporter | K02021 | Putative ABC transport system ATP-binding protein | Up |
| *SWI3D_ARATH* | Null | SWI SNF related, matrix associated, actin dependent regulator of chromatin, subfamily c, member | K11649 | SWI/SNF related-matrix-associated actin-dependent regulator of chromatin subfamily C | Up |
| *RH16_ARATH* | Helicase conserved C-terminal domain | Atp-dependent rna helicase | K14810 | ATP-dependent RNA helicase DDX56/DBP9 | Up |
| *MRG1_ARATH* | MRG | Mortality factor 4-like protein | K11339 | Mortality factor 4-like protein 1 | Up |
| *EPN1_ARATH* | ENTH domain | Clathrin interactor 1 | K12471 | Epsin | Up |
| *HAT22_ARATH* | Homodomain | Homeobox-leucine zipper protein | K01673 | Homeobox-leucine zipper protein | Up |
| *PDAT2_ARATH* | Lecithin: cholesterol acytransferase | Acyltransferase | K00679 | Phospholipid:diacylglycerol acyltransferase | Down |
| *SPSA4_ARATH* | Sucrose-6F-phosphate phosphohydrolase | Glycosyl transferase | K00696 | Sucrose-phosphate synthase | Down |
| *RPD5B_ARATH* | RNA polymerase Rpb5,  C-terminal domain | DNA-dependent RNA polymerase catalyzes | K03013 | DNA-directed RNA polymerases I, II, and III subunit RPABC1 | Down |
| *MYO12_ARATH* | Myosin head (motor domain) | Myosin heavy chain | K10357 | Myosin V | Down |
| *RL3_ORYSJ* | Ribosomal protein L3 | rRNA binding proteins | K02925 | Large subunit ribosomal protein L3e | Down |
| *UBQ11_ARATH* | Ubiquitin family | Ubiquitin | K08770 | Ubiquitin C | Down |
| *FAB1B_ARATH* | Phosphatidylinositol-4-phosphate 5-kinase | Phosphatidylinositol-4-phosphate 5-kinase | K00921 | 1-phosphatidylinositol-3-phosphate 5-kinase | Down |
| *RZ21A_ORYSJ* | RNA recognition motif. (RRM, RBD or RNP domain) | Serine arginine-rich splicing factor | K12896 | Serine/arginine-rich splicing factor 7 | Down |
| *CCX4_ARATH* | Null | Exchanger | K13754 | Solute carrier family 24 | Down |

Up represents upregulated gene in LPN compared to OPN; Down represents downregulated gene in LPN compared to OPN. Null represents unmatched result.
